# Long-term impact of 10-valent pneumococcal conjugate vaccine in Kenya: Nasopharyngeal carriage among children in a rural and an urban site six years after introduction

**DOI:** 10.1016/j.vaccine.2024.07.021

**Published:** 2024-11-14

**Authors:** Jennifer R. Verani, Daniel Omondi, Arthur Odoyo, Herine Odiembo, Alice Ouma, Juliet Ngambi, George Aol, Allan Audi, Samwel Kiplangat, Noel Agumba, Patrick K. Munywoki, Clayton Onyango, Elizabeth Hunsperger, Jennifer L. Farrar, Lindsay Kim, Miwako Kobayashi, Robert F. Breiman, Fabiana C. Pimenta, Maria da Gloria Carvalho, Fernanda C. Lessa, Cynthia G. Whitney, Godfrey Bigogo

**Affiliations:** a Respiratory Diseases Branch, Division of Bacterial Diseases, Centers for Disease Control and Prevention, 1600 Clifton Road, N.E. Atlanta, GA 30333, United States; b Division of Global Health Protection, Centers for Disease Control and Prevention, PO Box 606-00621, Village Market, Nairobi, Kenya; c Centre for Global Health Research, Kenya Medical Research Institute, P.O. Box: 1578 – 40100, Kisumu, Kenya; d Rollins School of Public Health, Emory University, 1518 Clifton Rd, Atlanta, GA 30322, United States; e Infectious Diseases and Oncology Research Institute, University of the Witwatersrand, 29 Princess of Wales Terrace, Johannesburg 2050, South Africa

**Keywords:** *Streptococcus pneumoniae*, Pneumococcal vaccines, Pneumococcal carriage, Child, Kenya

## Abstract

**Background:**

Kenya introduced Synflorix™ (GlaxoSmithKline, PCV10-GSK), a 10-valent pneumococcal conjugate vaccine, in 2011, using three primary doses and, in select areas, catch-up campaigns. Surveys conducted 1–2 years post-introduction showed a stable prevalence of pneumococcal colonization, with declines in vaccine-type carriage. However, little is known about the long-term impact of PCV10-GSK in Kenya.

**Methods:**

We conducted a cross-sectional survey of pneumococcal carriage among children aged <5 years in November–December 2017 in Kibera (Nairobi informal settlement, no catch-up) and Asembo (rural western Kenya, 2-dose catch-up for children 1–4 years), using the same methods and settings as prior annual surveys from 2009 to 2013. Participants were randomly selected from an ongoing population-based surveillance platform. Nasopharyngeal swabs were frozen in skim milk-tryptone-glucose-glycerin media within 4 h and underwent culture with broth enrichment for pneumococcus. Isolates were serotyped by polymerase chain reaction and Quellung.

**Results:**

We enrolled 504 children, including 252 from each site; >90 % of participants had received 3 doses of PCV10-GSK. Pneumococcal colonization was detected in 210 (83.3 %) participants in Kibera and 149 (59.1 %) in Asembo, which was significantly lower than the prevalence observed in 2013 (92.9 % and 85.7 %, respectively). PCV10-GSK serotypes were detected in 35/252 (13.9 %) participants in Kibera and 23/252 (9.1 %) in Asembo, respectively; these prevalences were lower, but not statistically different, from vaccine-type carriage prevalences in 2013 (17.3 % and 13.3 %, respectively). In 2017 in both sites, serotypes 3, 6A, 19A, 19F, and 35B were among the most common serotypes.

**Conclusion:**

Six years post-PCV10-GSK introduction, the prevalence of pneumococcal carriage among children has decreased, and the impact of PCV10-GSK on vaccine-type carriage has plateaued. Kenya recently changed from PCV10-GSK to Pneumosil™ (Serum Institute of India), a 10-valent PCV that includes serotypes 6A and 19A; these data provide historical context for interpreting changes in vaccine-type carriage following the PCV formulation switch.

## 1 Introduction

Global uptake of pneumococcal conjugate vaccines (PCVs) in routine infant immunization programs has led to major declines in pneumococcal pneumonia and invasive disease. Robust evidence of PCV impact is available from high-income settings, where PCVs were first introduced starting in 2000 [1], [2], [3]. Data on PCV impact have also accumulated from low- and middle-income settings [4], [5], [6], [7], where PCV introduction lagged by nearly a decade or more [8], and which suffer the greatest burden of pneumococcal disease [9], [10]. PCVs prevent disease both through direct protection of vaccinated individuals as well as indirect protection through decreased colonization with vaccine serotypes and subsequent decreased transmission in the community [11], [12], [13].

Following PCV introduction, prevalence of colonization with vaccine serotypes declines, while overall pneumococcal carriage prevalence generally remains stable, due to increases in carriage of non-vaccine serotypes [14], [15]. However, serotypes included in PCVs are those most prone to cause invasive disease; thus, declines in vaccine type carriage usually lead to net reduction in overall pneumococcal disease [16]. In the United States and Europe, vaccine-type colonization decreased dramatically following PCV introduction, leading to near elimination of circulation of vaccine serotypes in some settings [15]. Early evidence from several African counties also showed declines in vaccine-type carriage after PCV introduction, with reductions ranging from approximately 40 % to 75 % [17]. However the pre-PCV prevalence of vaccine-type carriage was higher in African countries than high-income settings [18]. One to two years after PCV introduction, approximately 10 to 20 % of healthy children were found to be colonized with vaccines serotypes, even in settings with high coverage and catch-up campaigns for children aged < 5 years [17]. Limited data are available on long-term impact of PCV in African countries, and it is unknown whether routine PCV use will lead to the extremely low levels of vaccine-type carriage observed in high-income settings.

Kenya introduced Synflorix™ (GlaxoSmithKline, PCV10-GSK), a 10-valent pneumococcal conjugate vaccine in January 2011, using a 3-dose schedule (6, 10, and 14 weeks) with no booster. Catch-up campaigns for children aged 1–4 years were conducted in select areas. Studies conducted one to two years post-introduction demonstrated reductions in vaccine-type carriage of approximately 50–60 % among children aged less than five years [19], [20]. We assessed the prevalence of vaccine-type carriage among healthy children 6 years post-PCV10-GSK introduction in two sites in Kenya where pre- and early post-PCV10-GSK carriage surveys had been carried out to better understand the long-term impact.

## 2 Methods

We conducted a community-based cross-sectional carriage survey among children aged < 5 years in two settings in Kenya using previously described methods [19]. Briefly, Kibera is a densely populated informal settlement located in Nairobi and characterized by crowded living conditions and poor sanitation. Asembo is a rural area in western Kenya that is sparsely populated with a high burden of malaria and HIV. PCV10-GSK was introduced in Asembo with a 2-dose catch up campaign for children aged 1–4 years at time of introduction, and with no catch up in Kibera.

The carriage survey was conducted using the same methods as prior annual pneumococcal carriage surveys from 2009 to 2013 [19]. Participants were randomly selected from children aged < 5 years enrolled in the Population–Based Infectious Disease Surveillance (PBIDS) platform, which monitors the health of an enumerated population of ∼ 25,000 individuals in Kibera and ∼ 35,000 persons in Asembo (as of mid-2017) [21]. In Asembo, PBIDS is nested within a larger Health and Demographic Surveillance (HDSS) platform [22]; in Kibera, PBIDS is a stand-alone platform. At the time of earlier cross-sectional carriage surveys, demographic data from PBIDS participants were collected during biweekly household visits. In 2015, the frequency of household visits decreased to two times per year; however, data collection tools remained unchanged. Criteria for being an active participant of PBIDS was residency in the study area for at least 4 months or being born to a parent who was a PBIDS participant, and consent provided by the head of household.

The target sample size for the 2017 cross-sectional survey was based on an expected reduction in the prevalence of vaccine-type pneumococcal carriage compared with the most recent cross sectional survey (2013), which showed a prevalence of 17.3 % and 13.3 % among children aged < 5 years in Kibera and Asembo, respectively [19]. Assuming 95 % confidence and 80 % power, a sample of 250 children in each site would provide sufficient power to detect a 50–60 % decline (50 % in Kibera and 60 % in Asembo) in vaccine-type pneumococcal carriage in children aged < 5 years in 2017 compared with 2013. In Asembo, we selected a simple random sample of children aged < 5 years from the HDSS/PBIDS database. In Kibera, consistent with prior surveys, we selected a stratified random sample from the PBIDS database, with 30 % of the sample selected from children < 12 months and 70 % from children aged 12–59 months. The oversampling of infants in Kibera was originally aimed at detecting early evidence of PCV impact among the vaccinated (since there was no catch-up in Kibera) and was carried forth through subsequent surveys to ensure consistency and comparability across surveys.

The survey was conducted during November–December 2017, the same time of year as prior surveys. Pre-selected participants were visited at home by trained community health workers to provide parents/guardians with information about the study; if interested, they were invited to present to a centrally located clinic in each site on a certain date. At the clinic, trained study staff obtained written informed consent, gathered demographic and epidemiologic data, and collected a single nasopharyngeal (NP) swab. Vaccination history was captured based on observation of the child health card; when the card was not available, we utilized vaccination data in the PBIDS database collected during routine household visits (also card-confirmed) or conducted follow-up visits to the household to obtain vaccine history if needed. NP swabs were placed in skim milk-tryptone-glucose-glycerin media and stored immediately in a cool box; they were vortexed and frozen and stored at −70◦C within 4 h.

All NP swabs were tested at the KEMRI-Centre for Global Health Research (CGHR) lab in Kisumu using identical methods as in 2009–2013 surveys [19]. Samples underwent broth enrichment culture (in Todd Hewitt broth with yeast extract and rabbit serum) for six hours before culturing for isolation in blood agar plates [23]. Optochin susceptibility and bile solubility were performed for any alpha-hemolytic isolates suspected to be *S. pneumoniae*. Pneumococcal isolates were serotyped by PCR at KEMRI-CGHR [24]; those not resolvable by PCR were sent to the *Streptococcus* Laboratory at the Centers for Disease Control and Prevention (CDC) in Atlanta for serotyping by Quellung. Additionally, all samples with no growth of pneumococcus and 10 % of those with pneumococcus isolated were sent to CDC for quality control. Susceptibility of pneumococcal isolates to commonly used antibiotics was performed at the CDC *Streptococcus* Laboratory using broth microdilution.

We calculated the percent of children aged < 12 months and aged 12–59 months with pneumococcal carriage and with carriage of PCV10-GSK serotypes (1, 4, 5, 6B, 7F, 9 V, 14, 18C, 19F, and 23F), using the Clopper-Pearson method to generate exact confidence intervals for binomial proportions [25]. We compared the prevalence of overall carriage and carriage of PCV10-GSK serotypes in 2017 to those observed in 2013 (2 years post-introduction) using the Fisher exact test and calculated percent change with 95 % confidence interval; data from carriage studies in 2009–2012 were also presented for context. We classified serotypes included in higher-valency PCVs including PCV13 (PCV10-GSK serotypes plus 3, 6A, and 19A), PCV15 (PCV13 serotypes plus 22F and 33F), and PCV20 (PCV15 serotypes plus 8, 10A, 11A, 12F, and 15B), as well as 10-valent Pneumosil™ (Serum Institute of India, PCV10-SII; serotypes 1, 5, 6A, 6B, 7F, 9 V, 14, 19A, 19F, and 23F). We compared the proportion of individual vaccine serotypes among all isolates in 2017 with 2013 using Fisher exact test. For antimicrobial susceptibility, we classified isolates as susceptible, intermediate, or resistant to specific antibiotics per 2018 Clinical & Laboratory Standards Institute guidelines; for penicillin we used both oral and parenteral breakpoints for non-meningitis syndromes [26].

The study protocol was reviewed by the U.S. CDC and was conducted consistent with applicable federal law and CDC policy§ (§See e.g., 45C.F.R. part 46, 21C.F.R. part 56; 42 U.S.C. §241(d); 5 U.S.C. §552a; 44 U.S.C. §3501 et seq). The protocol was also reviewed and approved by the KEMRI Scientific and Ethics Review Unit. Written informed consent was obtained from parents/guardians before enrollment.

## 3 Results

We enrolled a total of 504 eligible children, including (50.0 %) 252 from each site. Sixty-eight (27.0 %) in Kibera and 28 (11.1 %) in Asembo and were aged < 12 months. In both sites in 2017, >90 % of participants had received 3 doses of PCV10-GSK, a significant increase in coverage compared to 2013 (Table 1). In Kibera, significant declines were found between 2013 and 2017 in the prevalence of 4 or more people sleeping in the same room as the participant, cooking with wood or charcoal, cooking in the living/sleeping area, and recent antibiotic use; there were significant increases in the proportion of participants with cough and with fever in the past month and in PCV10-GSK coverage. In Asembo, compared to 2013, participants in 2017 less frequently reported 2 or more children in school/daycare in the household, cooking in the living/sleeping area, current cough and antibiotic use in the prior 7 days; they more frequently reported 4 or more people sleeping in the same room as the participant, current runny nose, fever in the past month, and diagnosis of pneumonia in the past month.

**Table 1:** Characteristics of children < 5 enrolled in cross sectional pneumococcal carriage surveys in 2013 and 2017.

Characteristic	Kibera			Asembo		
	2013 n = 462 n (%)	2017 n = 252 n (%)	p value	2013 n = 203 n (%)	2017 n = 252 n (%)	p value
Median age in months (IQR)	26 (9, 43)	24 (11,41)	0.9528	28 (18, 45)	32 (18, 46)	0.2374
Male sex	213 (46.1)	129 (51.2)	0.1935	103 (50.8)	120 (47.6)	0.5081
Number of PCV10 doses received						
0	109 (25.2)	0 (0)	<0.0001	8 (4.2)	1 (0.5)	<0.0001
1	11 (2.6)	2 (0.9)		21 (10.9)	3 (1.4)	
2	23 (5.3)	5 (2.3)		39 (20.2)	8 (3.9)	
3	289 (66.9)	209 (96.8)		125 (64.8)	195 (94.2)	
missing	30	36		10	45	
≥2 children in household attend primary school or daycare	264 (57.1)	153 (60.7)	0.3548	138 (68.0)	91 (36.1)	<0.0001
≥4 of people sleeping in same room with participant	395 (85.5)	162 (64.3)	<0.0001	61 (30.0)	111 (44.0)	0.0022
Smoker in household	44 (9.5)	18 (7.1)	0.2803	25 (12.3)	35 (13.9)	0.6219
Cook with wood/charcoal	436 (94.4)	175 (69.4)	<0.0001	202 (99.5)	249 (98.8)	0.4280
Cook in living/sleeping area	439 (95.0)	219 (86.9)	0.0001	45 (22.2)	34 (13.5)	0.0152
Current/recent illness						
Cough currently	157 (34.0)	68 (27.0)	0.0544	56 (27.6)	44 (17.5)	0.0095
Cough in past month	211 (45.8)	144 (57.1)	0.0037	81 (39.9)	102 (40.8)	0.8463
Runny nose currently	284 (61.5)	140 (55.6)	0.1240	68 (33.5)	107 (42.6)	0.0468
Fever in last 24 h	49 (10.6)	29 (11.5)	0.7193	23 (11.3)	38 (15.1)	0.2433
Fever in past month	154 (33.3)	103 (40.9)	0.0449	49 (24.1)	97 (38.6)	0.0010
Fast breathing in past month	44 (9.5)	30 (12.0)	0.3099	15 (7.4)	23 (9.2)	0.4973
Diagnosis of pneumonia in past month	20 (4.3)	10 (4.0)	0.8139	2 (1.0)	9 (3.8)	0.0606
Reported antibiotic use						
Within past 7 days	108 (23.4)	41 (16.3)	0.0255	42 (20.7)	29 (11.5)	0.0073
Within past month	195 (42.2)	86 (34.1)	0.0347	63 (31.0)	66 (26.2)	0.2545

**Fig. 1:**
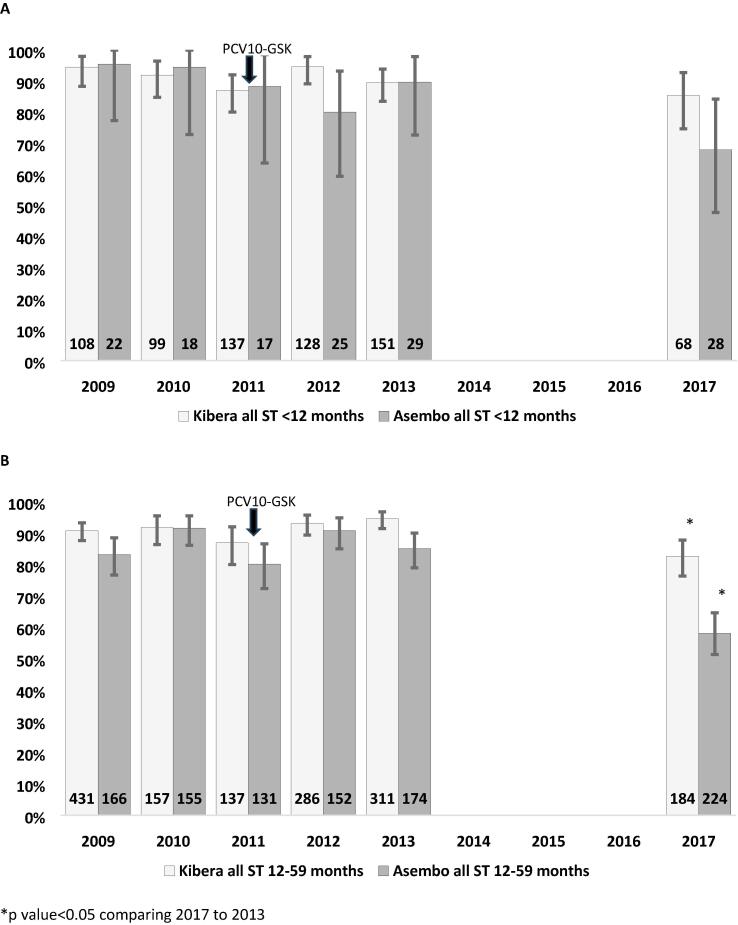
**Pneumococcal carriage among children aged < 12 months (A) and 12**–**59 months (B), Asembo and Kibera, Kenya, 2009**–**2013 and 2017.** Numbers at the base of each bar reflect number of participants. *p value < 0.05 comparing 2017 to 2013.

In 2017, pneumococcal colonization was detected in 210 (83.3 %) participants in Kibera and 149 (59.1 %) in Asembo. Within each site, the prevalence of colonization was significantly lower when compared with 2013, when 429/462 (92.9 %, p = 0.0001) in Kibera and 174/203 (85.7 %, p < 0.0001) in Asembo were colonized. Stratifying by age group and site (Fig. 1), we observed significant declines in overall pneumococcal carriage in the 12–-59-month age groups in both sites; changes in the < 12-month age group were not statistically significant.

**Fig. 2:**
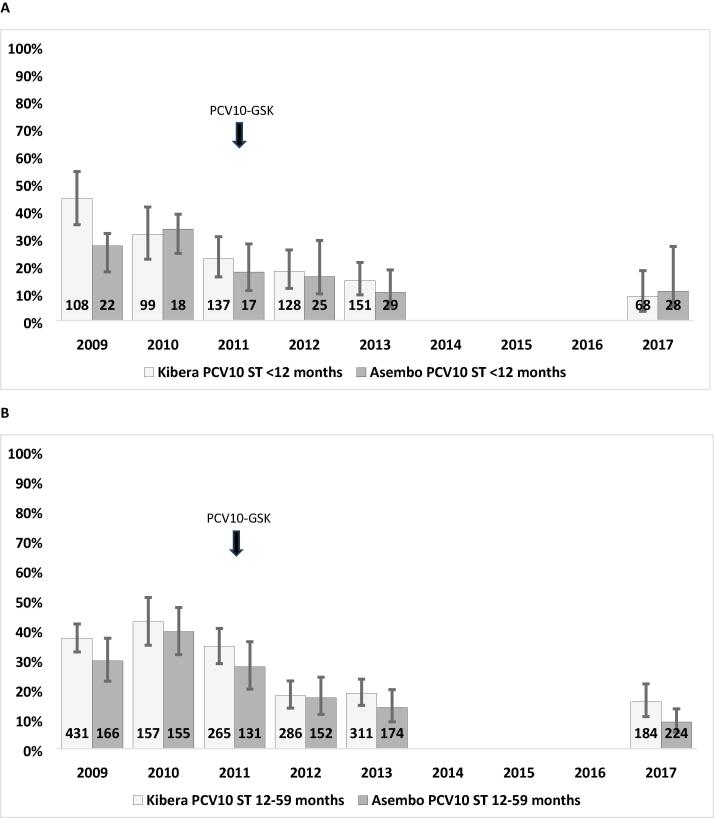
**Vaccine-type pneumococcal carriage among children < 12 months (A) and 12**–**59 months (B), Asembo and Kibera, Kenya, 2009**–**2013 and 2017.** Numbers at the base of each bar reflect number of participants.

In 2017, PCV10-GSK serotypes were detected in 35/252 (13.9 %) participants in Kibera and 23/252 (9.1 %) in Asembo, respectively. These prevalences of vaccine-type carriage were lower, but not statistically different, from those observed in 2013: 80/462 (17.3 %, p = 0.243) in Kibera and 27/203 (13.3 %, p = 0.176) in Asembo; expressed as a percent reduction, vaccine-type carriage declined by 19.6 % (95 % confidence interval [CI]: −27.1 %, 31.6 %) in Kibera and 31.4 % (95 %CI: –32.6 %, 39.4 %) in Asembo between 2013 and 2017. Although vaccine-type carriage declined between pre-PCV10-GSK period (2009 and 2010) and 2013 (Fig. 2), we observed no significant further decline between 2013 and 2017.

**Fig. 3:**
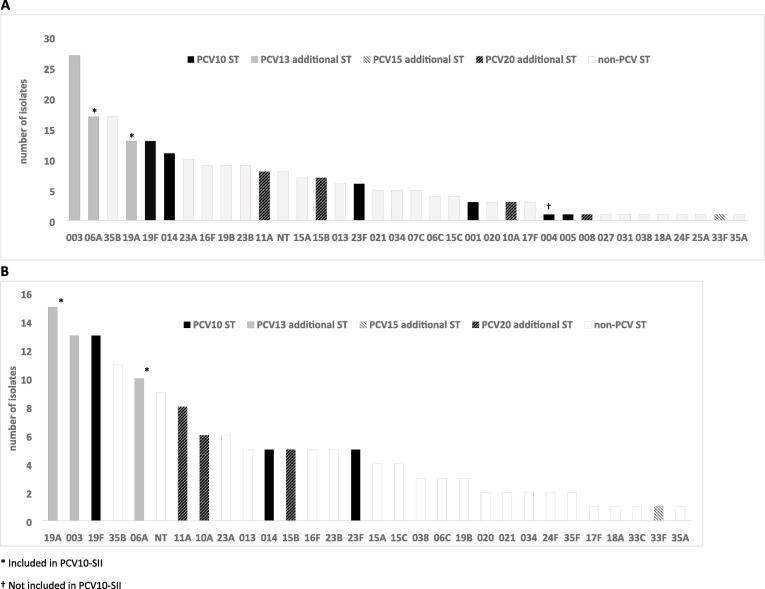
**Serotype frequency among carriage isolates from children aged < 5 years in Kibera (A, n = 223 isolates) and Asembo (B, n = 153 isolates), 2017** *Included in PCV10-SII. ^†^Not included in PCV10-SII.

**Fig. 4:**
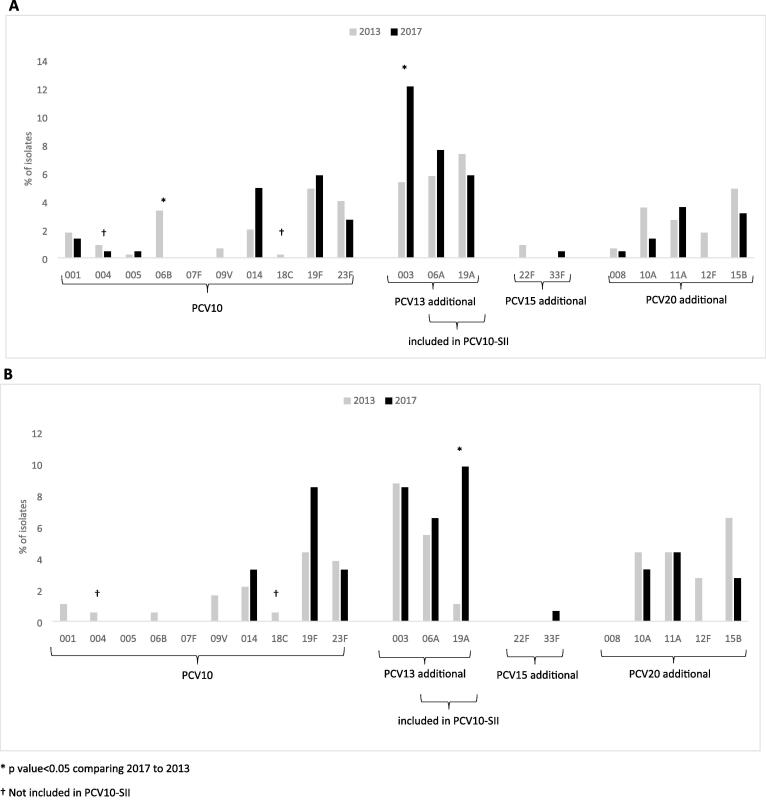
**Relative frequency of vaccine serotypes among carriage isolates from children aged < 5 years in Kibera in Kibera (A) and Asembo (B), in 2013 and 2017.** Kibera isolates were n = 450 in 2013 and n = 223 in 2017; Asembo isolates were n = 183 in 2013 and n = 153 in 2017. *Included in PCV10-SII. ^†^Not included in PCV10-SII.

Among 359 colonized children enrolled in 2017, 17 (4.7 %) had more than one serotype detected, yielding a total of 376 isolates (223 from Kibera and 153 from Asembo). In both sites, serotypes 3, 6A, 19A, 19F, and 35B were among the most common serotypes (Fig. 3). After 19F, the most frequently observed PCV10-GSK serotypes were 14 and 23F. Serotypes 1, 4, and 5 were detected in Kibera, but not in Asembo. Comparing the proportion of individual vaccine serotypes among all isolates in 2017 with 2013, significant changes included a decline in 6B (PCV10-GSK type; 0 % in 2017 vs. 3.3 % in 2013, p = 0.004) and an increase in 3 (PCV13 additional type; 12.1 % in 2017 vs. 5.3 % in 2013, p = 0.003) in Kibera and an increase in 19A (PCV13 additional and PCV10-SII type; 9.8 % in 2017 vs 1.1 % in 2013, p < 0.001) in Asembo (Fig. 4A, 4B). Among all isolates, the proportion of serotypes 14 and 19F (PCV10-GSK types), and serotype 6A (PCV13 additional and PCV10-SII type) rose in both sites, but the increase was not statistically significant.

Colonizing pneumococcal isolates were generally susceptible to penicillin based on parenteral breakpoints (Table 2). However, based on oral breakpoints, only 13.9 % of isolates in Kibera and 17.0 % in Asembo were fully susceptible to penicillin. No pneumococcal isolates were resistant to cefepime, cefotaxime, or ceftriaxone, and intermediate results on susceptibility testing for these antibiotics were rare. The prevalence of resistance to cefuroxime was 13.4 % among isolates from Kibera and 10.5 % among isolates from Asembo. Non-susceptibility to macrolides and clindamycin was significantly more common in Kibera than in Asembo. More than 90 % of isolates were resistant to trimethoprim sulfamethoxazole in both sites. No resistance to vancomycin, fluoroquinolones, or linezolid was detected.

**Table 2:** Antimicrobial susceptibility* of carriage isolates among children < 5 years, Kibera and Asembo, 2017.

Antibiotic class	Agent	Kibera (n = 223)			Asembo (n = 153)		
		Susceptible n (%)	Intermediate n (%)	Resistant n (%)	Susceptible n (%)	Intermediate n (%)	Resistant n (%)
Penicillins	Penicillin parental†	220 (98.7)	3 (1.4)	0 (0)	153 (1 0 0)	0 (0)	0 (0)
	Penicillin oral	31 (13.9)	162 (72.6)	30 (13.4)	26 (17.0)	114 (74.5)	13 (8.5)
	Amoxicillin Clavulanate†	222 (99.6)	1 (0.4)	0 (0)	152 (99.4)	0 (0)	1 (0.6)
Cephalosporins	Cefepime†	222 (99.6)	1 (0.4)	0 (0)	152 (99.4)	1 (0.6)	0 (0)
	Cefotaxime†	223 (1 0 0)	0 (0)	0 (0)	153 (1 0 0)	0 (0)	0 (0)
	Ceftriaxone†	220 (98.6)	3 (1.4)	0 (0)	152 (99.4)	1 (0.6)	0 (0)
	Cefuroxime parenteral	165 (74.0)	28 (12.6)	30 (13.4)	116 (75.8)	21 (13.7)	16 (10.5)
	Cefuroxime oral	193 (86.6)	0 (0)	30 (13.4)	137 (89.5)	0 (0)	16 (10.5)
Carbapenems	Meropenem	190 (85.2)	33 (14.8)	0 (0)	138 (90.2)	15 (9.8)	0 (0)
	Ertapenem	223 (1 0 0)	0 (0)	0 (0)	152 (99.4)	1 (0.6)	0 (0)
Glycopeptides	Vancomycin	223 (1 0 0)	0 (0)	0 (0)	153 (1 0 0)	0 (0)	0 (0)
Macrolides	Erythromycin	167 (74.9)	1 (0.4)	55 (24.7)	133 (86.9)	0 (0)	20 (13.1)
	Azithromycin	168 (75.3)	2 (0.9)	53 (23.8)	133 (86.9)	1 (0.9)	19 (12.4)
Tetracyclines	Tetracycline	139 (62.3)	5 (2.2)	79 (35.4)	110 (71.9)	8 (5.2)	35 (22.9)
Fluoroquinolones	Levofloxacin	223 (1 0 0)	0 (0)	0 (0)	153 (1 0 0)	0 (0)	0 (0)
	Moxifloxacin	223 (1 0 0)	0 (0)	0 (0)	153 (1 0 0)	0 (0)	0 (0)
Folate pathway antagonist	Trimethoprim Sulfamethoxazole	6 (2.7)	3 (1.4)	214 (96.0)	1 (0.6)	6 (3.9)	146 (95.4)
Phenicols	Chloramphenicol	208 (93.3)	0 (0)	15 (6.7)	149 (97.4)	0 (0)	4 (2.6)
Lincosamides	Clindamycin	198 (88.8)	0 (0)	25 (11.2)	144 (94.1)	9 (5.8)	0 (0)
Oxazolidines	Linezolid	223 (1 0 0)	0 (0)	0 (0)	153 (1 0 0)	0 (0)	0 (0)

*Based on 2018 Clinical and Laboratory Standards Institute (CLSI) breakpoints.

^†^Non-meningitis breakpoints used.

## 4 Discussion

Six years after PCV10-GSK introduction in Kenya, and in a setting with high vaccine coverage, we observed persistent circulation of vaccine serotypes. More than one in ten children aged less than 5 years were colonized with a PCV10 serotype. Despite an initial decline of ∼ 50–60 % in vaccine-type carriage observed among children aged less than 5 years in these study sites soon after PCV10-GSK introduction [19], and those lower levels of vaccine-type carriage were sustained through 2017. Vaccine-type invasive pneumococcal disease in Kenya also declined substantially following PCV10-GSK introduction without significant increase in non-vaccine-type disease [27]. However, between 2013 and 2017 we found no significant additional reduction in vaccine-type carriage despite increases in PCV10-GSK coverage, suggesting that vaccine impact on vaccine-type carriage plateaued. This pattern of continuing low-level circulation of vaccine serotypes is consistent with reports from other sub-Saharan African contexts [27], [28], [29], [30], and may be driven by a high force of infection [31]. In 2022, the Kenya National Vaccines and Immunization Program transitioned from PCV10-GSK to Pneumosil™ (Serum Institute of India, PCV10-SII), a 10-valent PCV with a slightly different serotype composition (includes 6A and 19A, does not include 4 and 18C) that was designed to be a more affordable PCV with optimized serotype coverage for high-burden areas [32]; the dosing schedule, 3 primary doses with no booster, remained unchanged. Additional assessments of pneumococcal carriage in Kenya will be important for understanding the extent to which vaccine serotypes continue to circulate and to monitor for potential resurgence of serotypes included in PCV10-GSK but not in PCV10-SII.

The prevalence of PCV10-GSK serotype carriage remained fairly stable between 2013 and 2017, with small, non-significant reductions. The only notable decline in a vaccine serotype was 6B in Kibera, which represented 3.3 % of colonizing isolates in 2013 but was not observed in 2017. The most common PCV10-GSK serotypes observed in 2017 were 19F, 14, and 23F; although 19F and 14 were relatively more common among colonizing isolates in 2017 compared with 2013, the changes were not significant. The persistence of serotypes 19F, 14, and 23F has also been observed in other sub-Saharan African settings using PCV10-GSK, including Nigeria [33] and Mozambique [34]. In 2017, we detected PCV10-GSK serotypes 1, 4, and 5 in Kibera, but infrequently (each < 2 % of all isolates); these serotypes were not observed in Asembo, highlighting the potential for geographic variability in circulating pneumococcal serotypes. Serotypes 3, 6A, 19A, and 35B were the most common non-PCV10-GSK serotypes in 2017. Three of these serotypes (3, 6A, and 19A) are included in all higher valent PCVs (PCV13, PCV15, and PCV20), and two (6A and 19A) are included in PCV10-SII, which recently replaced PCV10-GSK in Kenya. Regarding the two serotypes included in PCV10-GSK but not in PCV10-SII, in 2017 we observed only one child colonized with serotype 4 (in Kibera) and none colonized with serotype 18C. However, continued monitoring of circulating serotypes following the switch from PCV10-GSK to PCV10-SII is warranted.

Although vaccine-type carriage did not significantly decline from 2013 to 2017, we did observe an unexpected reduction in overall pneumococcal carriage. Typically, overall pneumococcal colonization remains stable after PCV introduction, while declines in vaccine-type carriage are offset by increases in non-vaccine-type carriage [14]. The reasons for the decline in overall colonization in both Asembo and Kibera are unclear. The sample collection and laboratory methods were unchanged, and the surveys were conducted during the same time of year. We did observe some changes in the study population between 2013 and 2017 with regard to factors known to be associated with pneumococcal colonization; however, the pattern was not consistent. For example, household crowding (defined for this analysis as ≥ 4 people sleeping the same room as the participant), which has been associated with pneumococcal carriage in a variety of settings [35], decreased in Kibera, but increased in Asembo. Cooking within the living/sleeping area decreased in both sites, which might have contributed to a decline in carriage [35]. However, reported recent antibiotic use also declined in both sites, which might be expected to lead to higher carriage prevalence. While the decline in pneumococcal carriage may be multifactorial, the lack of significant reduction in PCV10-GSK-type carriage during a time when overall carriage declined further suggests that PCV10 impact on carriage has plateaued.

The persistence of circulating vaccine serotypes in Kenya and other sub-Saharan Africa settings has raised questions about whether the PCV dosing schedule is optimized. PCVs are among the most expensive vaccines, so it is important to use doses as strategically and efficiently as possible. The World Health Organization currently recommends a 3-dose PCV schedule, with either three primary doses and no booster (3 + 0) or two primary doses and one booster dose at ≥ 9 months of age (2 + 1), with the choice of schedule taking into account programmatic considerations such as timeliness and coverage of routine infant immunizations [36]. Most countries in sub-Saharan Africa use a 3 + 0 schedule. However, data suggesting that a booster dose may be critical for controlling certain vaccine serotypes, including serotype 1, which is an important cause of invasive disease in sub-Saharan Africa (but rarely found in carriage), has prompted some African countries to switch from a 3 + 0 schedule to a 2 + 1 schedule [37]. This may be a consideration for Kenya and other settings where vaccine serotypes continue to circulate several years after PCV introduction.

Catch-up campaigns are thought to potentially accelerate the path towards herd protection from PCVs [37]. Although our study included an area with (Asembo) and without (Kibera) an initial catch-up campaign at the time of PCV10-GSK introduction, the sites are not directly comparable because they represent very different epidemiologic contexts and sampling in Kibera (consistent with methods of prior surveys) was skewed towards a younger median age. A modeling study using data from Kilifi, Kenya (another area with an initial PCV10 catch-up campaign) found that catch-up doses were an efficient way to achieve more quickly population-level protection against pneumococcal disease compared to vaccinating only infants [38]. Yet data from both Kilifi [27] and this study show a persistence of vaccine-type carriage (>5 % among children aged < 5 years) even after 5–6 years of PCV10-GSK implementation.

Antimicrobial-resistant *S. pneumoniae* poses an important global public health threat [39]. Among colonizing pneumococcal isolates in 2017, we found similar antimicrobial susceptibility patterns to what was observed in these same sites in 2013 [19]. The isolates were generally susceptible to penicillin using parenteral breakpoints, however, >80 % were non-susceptible to penicillin using oral breakpoints. Observed resistance to cefuroxime and persistence, albeit in low levels, of intermediate susceptibility to other cephalosporins highlights the need to continue monitoring pneumococcal resistance patterns to inform empiric treatment of respiratory infections.

This study is subject to several limitations. The data are from more than five years ago, so may not reflect the current prevalence of vaccine-type carriage, or the serotype distribution just prior to the switch from PCV10-GSK to PCV10-SII. The observed decline in overall pneumococcal carriage between 2013 and 2017 complicates the interpretation of PCV10-GSK impact on vaccine-type carriage during that time period, since other non-PCV factors have likely influenced both outcomes. The use of different sampling schemes (simple random sample of all children under age 5 years in Asembo and oversampling of infants in Kibera) limits the comparability of the data across sites and skews the prevalences observed in Kibera towards those of the infants. Recent antibiotic use was lower in 2017 than in 2013, but still fairly common; this could affect both carriage prevalence and antimicrobial susceptibility patterns. While the study was powered to detect a 50–60 % reduction in vaccine-type carriage between 2013 and 2017, it is possible that smaller declines occurred, and the study was underpowered to detect them.

Although PCV10-GSK introduction in Kenya led to a substantial reduction in vaccine-type carriage in the 2–3 years following introduction, that trend plateaued over the subsequent 3–4 years. In 2017, PCV10-GSK serotypes including 19F, 14, and 23F continued to commonly circulate in Kenya. Three of the most frequently carried non-PCV10-GSK serotypes (3, 6A, and 19A) are included in higher valent PCVs, and two (6A and 19A) are included in PCV10-SII, which recently replaced PCV10-GSK in the Kenyan routine infant immunization program. These data provide historical context for interpreting any changes in vaccine-type carriage following the PCV formulation switch and provide further evidence that a booster-dose containing schedule (i.e., 2 + 1) may be needed to optimize PCV impact on the circulation of vaccine serotypes in high-burden settings.

## CRediT authorship contribution statement

**Jennifer R. Verani:** Conceptualization, Data curation, Formal analysis, Funding acquisition, Investigation, Methodology, Project administration, Resources, Supervision, Visualization, Writing – original draft, Writing – review & editing. **Daniel Omondi:** Data curation, Formal analysis, Investigation, Project administration, Supervision, Writing – review & editing. **Arthur Odoyo:** Data curation, Investigation, Writing – review & editing. **Herine Odiembo:** Data curation, Investigation, Writing – review & editing. **Alice Ouma:** Conceptualization, Methodology, Project administration, Supervision, Writing – review & editing. **Juliet Ngambi:** Investigation, Methodology, Writing – review & editing. **George Aol:** Conceptualization, Methodology, Project administration, Supervision, Writing – review & editing. **Allan Audi:** Conceptualization, Data curation, Methodology, Software, Supervision, Validation, Writing – review & editing. **Samwel Kiplangat:** Data curation, Methodology, Validation, Writing – review & editing. **Noel Agumba:** Data curation, Methodology, Validation, Writing – review & editing. **Patrick K. Munywoki:** Methodology, Supervision, Validation, Visualization, Writing – review & editing. **Clayton Onyango:** Resources, Supervision, Writing – review & editing. **Elizabeth Hunsperger:** Resources, Supervision, Writing – review & editing. **Jennifer L. Farrar:** Conceptualization, Methodology, Resources, Writing – review & editing. **Lindsay Kim:** Conceptualization, Data curation, Methodology, Resources, Writing – review & editing. **Miwako Kobayashi:** Data curation, Resources, Writing – review & editing. **Robert F. Breiman:** Conceptualization, Methodology, Resources, Writing – review & editing. **Fabiana C. Pimenta:** Data curation, Investigation, Validation, Writing – review & editing. **Maria da Gloria Carvalho:** Conceptualization, Data curation, Investigation, Methodology, Validation, Writing – review & editing. **Fernanda C. Lessa:** Conceptualization, Funding acquisition, Methodology, Resources, Supervision, Writing – review & editing. **Cynthia G. Whitney:** Conceptualization, Methodology, Software, Supervision, Writing – review & editing. **Godfrey Bigogo:** Conceptualization, Funding acquisition, Methodology, Project administration, Resources, Supervision, Writing – review & editing.

## Declaration of competing interest

The authors declare that they have no known competing financial interests or personal relationships that could have appeared to influence the work reported in this paper.

## Data Availability

Data will be made available on request.
